# Testing the Role of Glutamate NMDA Receptors in Peripheral Trigeminal Nociception Implicated in Migraine Pain

**DOI:** 10.3390/ijms23031529

**Published:** 2022-01-28

**Authors:** Cindy Guerrero-Toro, Kseniia Koroleva, Elizaveta Ermakova, Oleg Gafurov, Polina Abushik, Pasi Tavi, Guzel Sitdikova, Rashid Giniatullin

**Affiliations:** 1 A. I. Virtanen Institute for Molecular Sciences, University of Eastern Finland, 70211 Kuopio, Finland; cindyg@uef.fi (C.G.-T.); k.s.koroleva@yandex.ru (K.K.); polinaabushik@gmail.com (P.A.); pasi.tavi@uef.fi (P.T.); 2 Department of Human and Animal Physiology, Institute of Fundamental Medicine and Biology, Kazan Federal University, 420008 Kazan, Russia; latinochrome0@gmail.com (E.E.); gsholeg@gmail.com (O.G.); 3 Laboratory of Comparative Neurophysiology, Sechenov Institute of Evolutionary Physiology and Biochemistry, Russian Academy of Sciences, 194223 Saint Petersburg, Russia

**Keywords:** glutamate, migraine, NMDA, CGRP, trigeminal ganglia, trigeminal nerve

## Abstract

The pro-nociceptive role of glutamate in the CNS in migraine pathophysiology is well established. Glutamate, released from trigeminal afferents, activates second order nociceptive neurons in the brainstem. However, the function of peripheral glutamate receptors in the trigeminovascular system suggested as the origin site for migraine pain, is less known. In the current project, we used calcium imaging and patch clamp recordings from trigeminal ganglion (TG) neurons, immunolabelling, CGRP assay and direct electrophysiological recordings from rat meningeal afferents to investigate the role of glutamate in trigeminal nociception. Glutamate, aspartate, and, to a lesser extent, NMDA under free-magnesium conditions, evoked calcium transients in a fraction of isolated TG neurons, indicating functional expression of NMDA receptors. The fraction of NMDA sensitive neurons was increased by the migraine mediator CGRP. NMDA also activated slowly desensitizing currents in 37% of TG neurons. However, neither glutamate nor NMDA changed the level of extracellular CGRP. TG neurons expressed both GluN2A and GluN2B subunits of NMDA receptors. In addition, after removal of magnesium, NMDA activated persistent spiking activity in a fraction of trigeminal nerve fibers in meninges. Thus, glutamate activates NMDA receptors in somas of TG neurons and their meningeal nerve terminals in magnesium-dependent manner. These findings suggest that peripherally released glutamate can promote excitation of meningeal afferents implicated in generation of migraine pain in conditions of inherited or acquired reduced magnesium blockage of NMDA channels and support the usage of magnesium supplements in migraine.

## 1. Introduction

Migraine is a common and complex neurological disorder, which neurochemical mechanisms leading to a severe headache are largely unknown [1,2]. Glutamate, a major excitatory neurotransmitter in the central nervous system [3], mediates the synaptic transmission from the primary afferents to the second order nociceptive neurons in the brainstem [4]. Glutamate also plays a key role in cortical hyperexcitability in familial type migraine [5]. Peripheral nerves release glutamate in response to thermal or electrical stimulation [6,7]. However, the role of glutamate in peripheral mechanisms of migraine, in particular, in the generation of nociceptive pain signals in meninges, which are supposed to be origin site for migraine pian, remains poorly understood. In general, glutamate mediates its physiological functions acting via ionotropic α-amino-3-hydroxy-5-methyl-4-isoxazolepropionic acid (AMPA), N-methyl-D-aspartate (NMDA) or kainate receptors to depolarize neurons, whereas metabotropic (mGluR) glutamate receptors can modulate neuronal activity, thus contributing to neuropathic or inflammatory pain processes [8,9,10,11,12].

In migraine models, the antagonists of NMDA and AMPA but not kainate receptors prevented the dilatory action of the migraine-related neuropeptide CGRP on rat dural artery [13]. Kynurenate, which is a nonspecific endogenous antagonist of NMDA, AMPA and kainate receptors, can block the release of CGRP [14] suggesting the role of these receptors in meningeal pro-inflammatory and pro-nociceptive processes. Moreover, the expression of GluN1, GluN2A, and GluN2B subunits of NMDA receptor has been demonstrated in rat trigeminal ganglia (TG) [15,16,17,18]. GluN2B subunits of NMDA receptors were found to be expressed in nerve fibers innervating dural blood vessels [19]. TG neurons were excited by the direct NMDA injection to the ganglion [20] or by stimulation of the peripheral receptive field in the face [16,20,21,22,23]. However, the functional responses of trigeminal afferent fibers in the meninges to the specific NMDA agonists were not described.

Notably, NMDA receptors, which are of special interest in migraine [15,17,18,24], are normally blocked by magnesium ions and this block can be relieved by local depolarizing agents [25]. Interestingly, magnesium-deficient rats develop a mechanical hyperalgesia [26]. Several studies have revealed that the level of serum magnesium was lower in migraine patients than in non-migraineurs and related to the frequency of migraine attacks [27,28]. On other hand, glutamate concentrations and expression levels of GluN2B subunits of NMDA receptors were higher in migraine patients, but declined during migraine treatment [24]. Expression of NMDA receptors is sensitive to inflammatory processes [29] which can be triggered by the neuropeptide CGRP which plays a key role in migraine promoting neurogenic inflammation in the trigeminovascular nociceptive system [30,31,32,33]. However, the action of CGRP on NMDA receptors and action of NMDA on CGRP release from trigeminal neurons were not yet studied.

Clinical observations on the promotion of headache by large ingestion of monosodium glutamate [34] also suggest the peripheral site of action as this compound does not cross the blood brain barrier [35]. Consistent with this, systemic administration of monosodium glutamate in rats induced sensitization to mechanical stimuli and increased the firing of neurons in the spinal trigeminal subnucleus caudalis (SpVc) [19]. However, a direct testing of glutamate action on meningeal trigeminal afferents which are different in functional properties from somas of TG neurons [36] was not performed to date.

In the present study, we explored the presence and functional expression of glutamate NMDA receptors in somas of TG neurons and in meningeal trigeminal nerve terminals. We also tested whether the activity of these receptors could be modulated by the neuropeptide CGRP, and if glutamate and NMDA promote the release of CGRP from the trigeminal ganglion cells.

## 2. Results

### 2.1. Effect of Glutamate, Aspartate, and NMDA on Intracellular Calcium Level in Trigeminal Ganglion Neurons

First, we tested at the level of single cells, whether TG neurons express functional glutamate receptors. To this end, we used calcium imaging technique which allows to characterize glutamate receptors in a large population of neurons. Neurons were distinguished from other cells in culture, based on responses to KCl [37].

**Figure 1:**
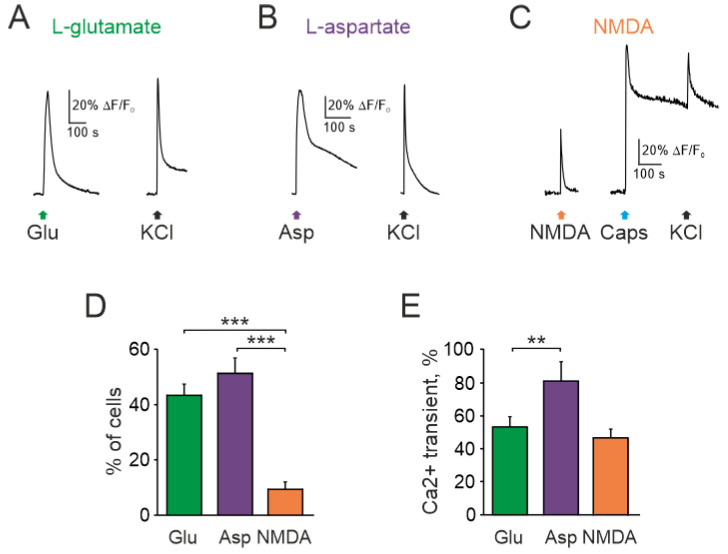
Intracellular Ca^2+^ transients activated by glutamate receptors agonists in rat TG neurons: Representative Ca^2+^ traces recorded after (**A**) glutamate (1 mM), (**B**) aspartate (100 µM), and (**C**) NMDA (100 µM) applications to TG neurons. All agonists were mixed with the co-agonist glycine (10 µM) in magnesium free solution. Average of 5 traces in each. Notice that NMDA sensitive neuron also responded to the TRPV1 agonist capsaicin; (**D**) Histograms showing the percentage of neurons responding to three glutamate agonists. Notice that the number of neurons responding to NMDA was significantly less than to glutamate and aspartate; (**E**) Histograms showing amplitudes of Ca^2+^ transients (normalized to ionomycin response) activated by glutamate, aspartate, NMDA. All agonists were applied with the co-agonist glycine. Mean ± SEM. ** *p* < 0.01; *** *p* < 0.001.

Application of 1 mM glutamate with the co-agonist glycine (10 µM) for 2 s, in magnesium free solution, induced calcium transients in a fraction of TG neurons. Figure 1A–C shows representative traces of calcium responses in neurons. Totally, 44% of neurons (68/156) responded to glutamate (*n* = 7, Figure 1A,D). Likewise, the other broad-spectrum agonist aspartate (100 µM) with co-agonist glycine (10 µM, 20 s) activated similar number of neurons (51%, 40/78, Figure 1B,D) with larger amplitude of responses to aspartate (53.9 ± 5.3%, 68 cells for glutamate vs. 81.3 ± 11.1%, 40 cells for aspartate, *p* < 0.01, Figure 1E).

As high affinity glutamate-NMDA receptors are implicated in peripheral glutamatergic nociception [18,21,23,38,39], next we tested the action of NMDA (100 µM with co-agonist glycine, 10 µM). In contrast to glutamate and aspartate, only a small fraction of neurons generated calcium transients to NMDA (12/124, *n* = 7, Figure 1C,D). This value (9.6%) was clearly lower than glutamate and aspartate responsiveness of TG neurons.

**Figure 2:**
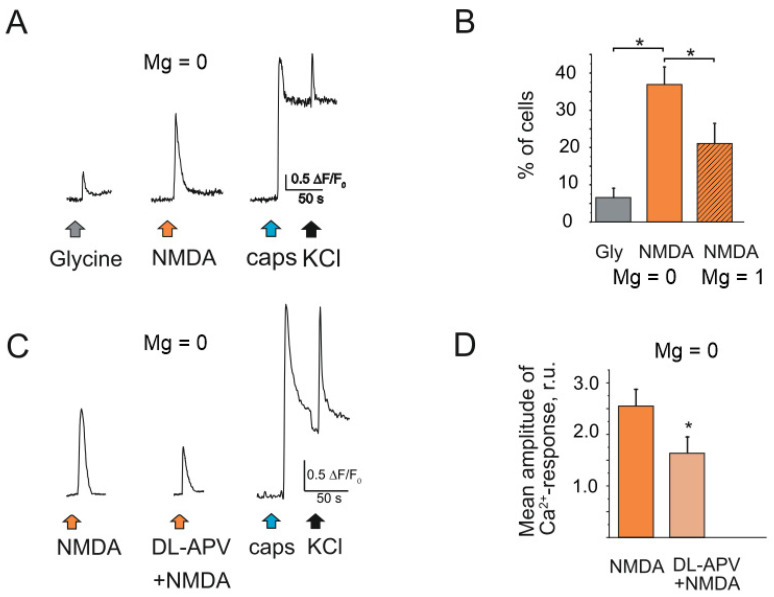
Intracellular Ca^2+^ transients activated by NMDA in rat TG neurons: (**A**) Representative Ca^2+^ traces recorded after glycine (30 µM) and NMDA (100 µM) applications to TG neurons. All agonists were in magnesium free solution. NMDA sensitive neuron also responded to the TRPV1 agonist capsaicin; (**B**) Histograms showing the percentage of neurons responding to glycine and NMDA in magnesium free solution vs. NMDA in basic solution (Mg 1 mM); (**C**) Representative Ca^2+^ traces recorded after NMDA (100 µM) and combination of DL-APV (80 µM) + NMDA applications to TG neurons. All drugs were in magnesium free solution. (**D**) Histograms showing the mean amplitude of Ca^2+^ -response activated by NMDA and against the background of DL-APV. Mean ± SEM. * *p* < 0.05.

The increase of glycine concentration up to 30 µM elevated the fraction of neurons responded to NMDA to 36.9 ± 4.5% (41/112 cells, *n* = 3, Figure 2A,B). At the same time only 6.6 ± 2.4% of cells were activated by solely glycine application (8/112 cells, *n* = 3, *p* < 0.05). The presence of magnesium in extracellular solution decreased the fraction of responding cells to 20.6 ± 5.2% (32/160 cells, *n* = 3, *p* < 0.05). The amplitude of NMDA evoked Ca^2+^ transients were decreased to 60.1 ± 6.8% by the specific antagonist of NMDA receptor DL-APV (80 µM) (*n* = 3, Figure 2C,D).

### 2.2. Immunolabeling NMDA Receptors in Trigeminal Ganglion Neurons

**Figure 3:**
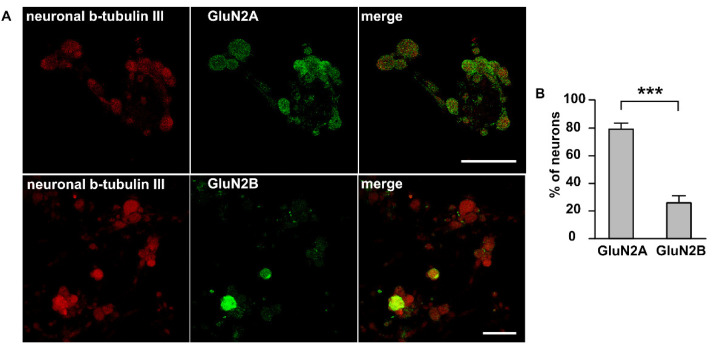
Immunolabeling NMDA receptors in trigeminal ganglion neurons: (**A**) Immunostaining of GluN2A and GluN2B subunits of NMDA receptor in TG cells; Left column—labelling of β-tubulin III; central column—labelling with GluN2A (top) or GluN2B (bottom) antibodies; right column—overlay. Representative images of the staining are made at original magnification 63 × 1/4. Scale bar: 50 μM (**B**) Histogram presented the percentage of neurons expressed GluN2A and GluN2B subunits, *** *p* < 0.001.

To identify a general pool of NMDA receptors in TG neurons we used an immunocytochemical detection of two major subtypes (synaptic GluN2A and extrasynaptic GluN2B) of NMDA receptors in isolated TG culture. Interestingly, this staining showed significant differences between GluN2A and GluN2B subunits profiles. GluN2A subunits were highly expressed in a dotty manner in significant number of neuronal cell bodies marked by the neuronal β-tubulin III staining (Figure 3A, red). In general, 80 ± 3.7% of trigeminal cells expressed GluN2A subunits (458 cells, *n* = 4). Unlike GluN2A, GluN2B subunits were expressed in more limited fraction of TG neurons (26.7 ± 4.5% of β-tubulin III positive cells, 311 cells, *n* = 4, Figure 3B).

Thus, immunolabelling revealed even a large presence of two major subtypes of NMDA receptors in TG neurons.

### 2.3. CGRP Increases NMDA Responding TG Cells

**Figure 4:**
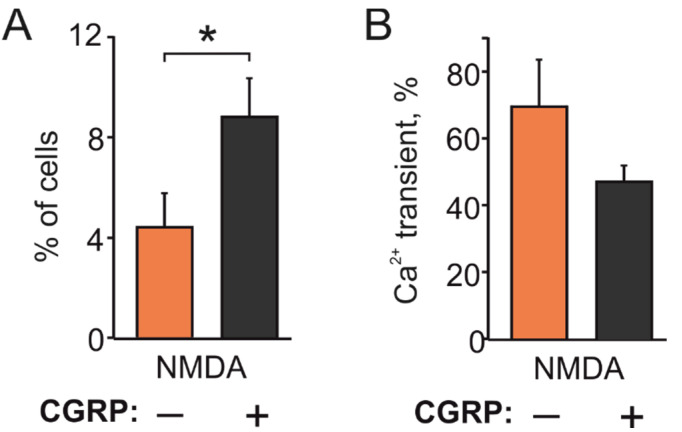
Facilitating effect of CGRP on NMDA evoked Ca^2+^ responses: (**A**) Histograms showing that CGRP (1 µM, 2 h) increased the fraction of TG neurons responding to NMDA (100 µM) with the co-agonist glycine (10 µM) in P12-14 rats (247 neurons in control; 318 neurons after CGRP); (**B**) Histograms showing amplitudes of calcium responses in TG neurons responding to NMDA. Notice that the amplitudes were not significantly changed (*p* > 0.05). Mean ± SEM. * *p* < 0.05.

As the neuropeptide CGRP plays a key role in the pathophysiology of migraine [32,40], we next tested the action of CGRP on NMDA-induced responses. To determine whether the migraine mediator CGRP can increase responses to NMDA, we pre-incubated TG cultures obtained from P12 rats with 1 µM CGRP for 2 h. In control, in these experiments, 11/247 cells responded to NMDA (*n* = 7). However, after CGRP exposure, the number of responding neurons was almost doubled (28/318 cells, *n* = 7, *p* < 0.05, Figure 4A), with a non-significant decrease in amplitude (69.4 ± 14.6% in control vs. 47.6 ± 4.7% after CGRP, *p* > 0.05, Figure 4B).

Taken together, these data indicated that TG neurons express various subtypes of glutamate receptors including NMDA receptor subtype and that the fraction of NMDA positive neurons could be enhanced in the presence of CGRP for 2 h.

### 2.4. NMDA Currents in Trigeminal Ganglion Neurons

**Figure 5:**
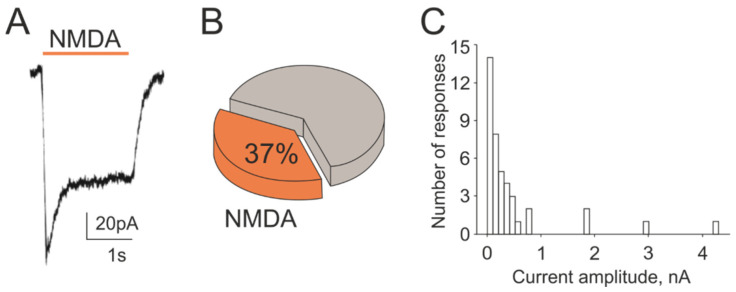
NMDA-evoked membrane currents in rat TG neurons: (**A**) Representative membrane current traces activated by NMDA (100 µM, 2 s) mixed with co-agonist glycine (30 µM) recorded in magnesium-free solution from TG neurons at the holding potential −40 mV; (**B**) Percentage of responding cells induced by NMDA from all recorded neurons (110 cells); (**C**) Distribution of peak amplitudes of NMDA evoked currents in TG neurons.

As the independent and direct approach to characterize glutamate responses in TG neurons, we used patch clamp recordings of membrane currents from these cells activated by the local application of NMDA. Application of NMDA (100 µM with glycine 30 µM in magnesium free solution) on TG neurons induced membrane currents in 37% of tested cells (from 110 cells, *n* = 12, Figure 5A,B). Notably, the amplitude of NMDA currents was very variable ranging from 20 pA to 4 nA (Figure 5C). Thus, in 14 cells of NMDA responsive neurons, membrane currents had the amplitude lower than 100 pA and only in 4 of all tested neurons) of cells they exceeded 1000 pA. These data confirmed functional expression of NMDA receptors in significant number of TG neurons.

### 2.5. Action of Glutamate and NMDA on CGRP Release

**Figure 6:**
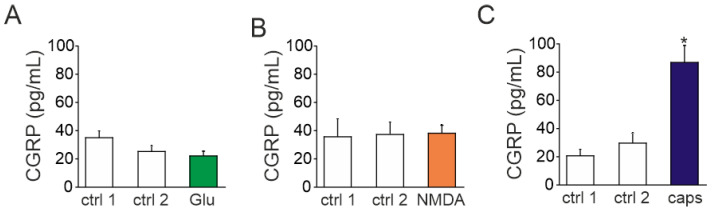
CGRP release by NMDA, glutamate, and capsaicin in TG cells: (**A**) Histogram showing CGRP concentration in control (ctrl) and after application of glutamate (Glu, 1 mM with 10 µM glycine) for 15 min to TG cells obtained from P10-P12 rats. Notice that CGRP level was not changed by glutamate application; (**B**) CGRP concentration in control and after exposure to NMDA (30 µM with 10 µM glycine) for 15 min. Like with glutamate, after application of NMDA, the level of CGRP remained at a basal level; (**C**) CGRP concentration after exposure to capsaicin (1 µM), positive control. Notice large increase in CGRP release. Mean ± SEM. * *p* < 0.05.

Given the key role of CGRP in migraine, we next tested whether activation of glutamate receptors can trigger the release of this migraine mediator from the fraction of peptidergic TG neurons. To this end, we used an enzymatic immune assay (EIA) to detect CGRP released into medium of TG cultures prepared from P12 rats. Samples were sequentially collected in duplicates from well plates in control and after exposure to glutamate agonists. Exposure to 1 mM glutamate did not change the release of CGRP (21.7 ± 3.5 pg/mL, *n* = 10, Figure 6A) comparing to control (25 ± 4.3 pg/mL, *p* > 0.05). Similar results were obtained with 100 µM NMDA combined with 10 µM glycine (37.8 ± 12.2 pg/mL in control vs. 38.1 ± 5.8 pg/mL after NMDA with glycine, *n* = 9, *p* > 0.05, Figure 6B). In contrast, the potent TRPV1 agonist capsaicin (1 µM), used as a positive control, induced a large CGRP release (89.9 ± 11.7 pg/mL, *n* = 4, vs. 29 ± 7.4 pg/mL in control, *p* < 0.05, Figure 6C). Thus, despite the presence of glutamate receptors in neurons, the respective agonist of these receptors did not promote CGRP release.

### 2.6. NMDA Induced Nociceptive Activity in Trigeminal Nerves in the Absence and Presence of Magnesium Ions

**Figure 7:**
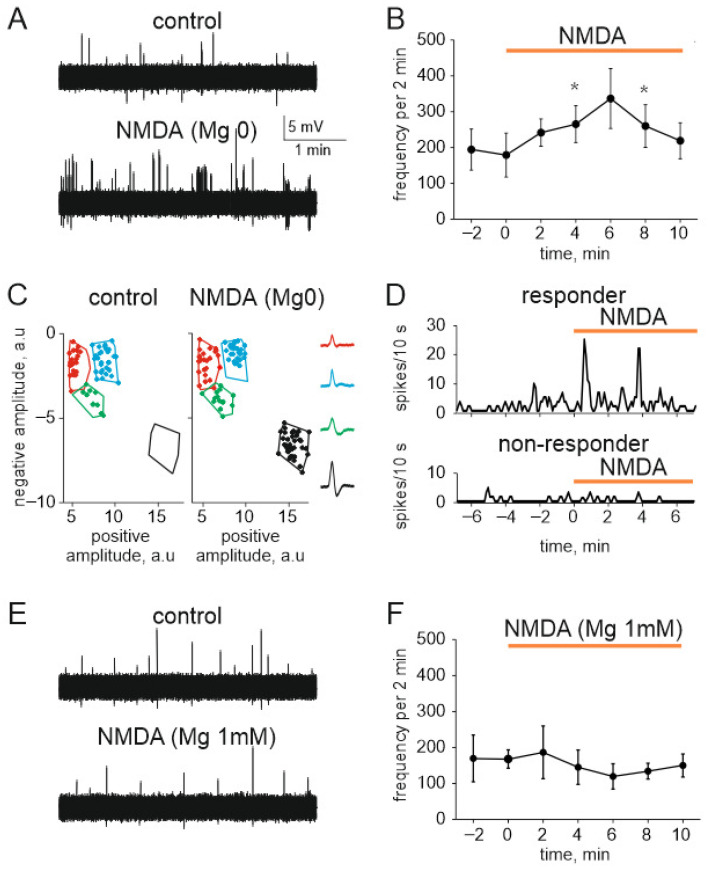
NMDA increases nociceptive spiking in meningeal trigeminal afferents: (**A**) Sample traces of spontaneous multiple unit activity (MUA) in control (top) and in the presence of NMDA (100 μM with 30 µM glycine) in magnesium free solution (bottom); (**B**) The time-course of frequency of nociceptive spikes (2 min binning) during 4 min recording in control (before agonist) and during application of NMDA (100 µM, 10 min); (**C**) Cluster analysis of nociceptive spikes in control and in the presence of NMDA. Spike’s positive phase amplitudes (abscissa) are plotted vs. negative phase amplitudes in arbitrary units (a.u.) (ordinate) to confirm clusters compactness. Color contours outline spike clusters separated by KlustaKwik method. Notice that green MUA increased in numbers whereas black dots (initially the silent cluster) appeared during NMDA application. Insets show average shapes; (**D**) Example of the time-course of spike frequency for the responder and non-responder clusters before and after NMDA application; (**E**) Sample traces of spontaneous MUA in control (top) and in the presence of NMDA (100 μM with 30 µM glycine) in basic ACSF solution (bottom); (**F**) The time-course of frequency of nociceptive spikes (2 min binning) during 4 min recording in control (before agonist) and during application of NMDA (100 µM, 10 min) in basic ACSF solution. * *p* < 0.05.

Finally, to test the role of peripheral NMDA receptors in more physiologically relevant conditions we used the whole-mount hemiskull preparation with preserved meningeal innervation with intact peripheral branches of the trigeminal nerve [36]. Figure 7A shows an example of nociceptive activity in meningeal afferents before (in magnesium-free solution supplemented by 30 µM glycine) and after application of 100 μM NMDA with glycine (30 µM). The global spiking activity of the whole nerve was significantly increased from 179 ± 61 spikes in 2 min before NMDA application (*n* = 5) to 265 ± 51 and 260 ± 59 spikes by the fourth and eighth minutes of NMDA application, respectively (*n* = 5, *p* < 0.05, Figure 7B). Using our novel clustering approach [36,41,42] we performed the neurochemical profiling of nociceptive spiking in different fibers of meningeal afferents. This approach revealed sensitive to NMDA application fibers (so-called ‘responder clusters’) and ‘non-responders’ [36] (Figure 7C). Interestingly, there were clusters entirely inactive in control which became very active in the presence of NMDA (Figure 7D). We found 13 such ‘sleeping’ clusters which were activated only after application NMDA characterized by relatively large amplitude of spikes (Figure 7C, right). In general, in five experiments, 32 ± 3% of clusters showed sensitivity (>2-fold increase in mean frequency) to NMDA application.

Next, to explore the functional role of NMDA receptors in non-sensitized conditions with preserved stable block of these receptors by magnesium ions, we performed testing of the action of NMDA on meningeal nerve spikes in the presence of 1 mM magnesium (Figure 7E). As expected from the blocking role of magnesium ions on NMDA receptor, we did not find significant change of nociceptive activity in response to NMDA application (*n* = 4; Figure 7F).

In summary, we found that the very peripheral nerve branches of the trigeminal nerve in meninges are equipped by the functional magnesium-sensitive NMDA receptors, and they are differentially presented in individual nerve fibers.

## 3. Discussion

The main finding of the current study is the demonstration, by using various approaches, the presence of functional glutamate NMDA receptors in somas of TG neurons and in meningeal peripheral terminals of trigeminal nerve implicated in generation of migraine pain. The excitatory action of NMDA was evident in conditions of reduced magnesium blockage of this receptor type which mimics conditions of magnesium deficiency observed in fraction of migraine patients. Our data also show for the first time that TG neurons are sensitive to the endogenous aminoacid aspartate. In addition, we found that the pro-nociceptive signaling by NMDA in neurons can be enhanced in the presence of CGRP. Thus, apart from the well-established role of glutamate in transmission of nociceptive signals from primary afferents to brainstem neurons and cortical excitability in migraine, we show that the activation of NMDA receptors in tissues located outside of the brain-blood barrier can potentially contribute to peripheral pain-triggering mechanisms of migraine.

Using the broad-spectrum glutamate receptor agonists, glutamate and aspartate, we showed here with a calcium imaging approach that the essential fraction of TG neurons responds to these endogenous agonists. Interestingly, in neurons, we observed responses to glutamate agonists even without using an inhibitor of glutamate uptake [20], which blocks glutamate transporter in glial cells and decreases extracellular glutamate level [43] through the glutamate-glutamine cycle [20,44]. Our main focus, in the current study, was on the role of calcium-permeable NMDA receptors. With calcium imaging we found that the number of responded to NMDA cells was lower than fraction of neurons responding to glutamate and aspartate. This is not surprising as glutamate can act on ionotropic AMPA, NMDA, kainate and various subtypes of metabotropic receptors. The nature of other than NMDA glutamate receptors in these cells requires future experiments. Nevertheless, our patch clamp approach revealed that one third of neurons express functional NMDA receptors mainly giving rise for low amplitude membrane currents. A higher total fraction of responding neurons was detected by immunolabeling detecting the presence of a large fraction of GluN2A receptor subunits in TG neurons.

Previous studies demonstrated RNA and protein expression of GluN1, GluN2A and GluN2B subunits of NMDA receptor in TG tissues [15,17,18,20]. While the GluN1 subunits were expressed in 99% of ganglionic neurons [18], GluN2A and GluN2B immunoreactivity was present in a limited fraction of cells [17,18]. Thus, about 30% of TG neurons innervating the TMJ or masseter muscle contained GluN2B subunits [17,18], which is close to values obtained in our study. However, expression of the GluN2A subunit in our study was much higher (~80%) compared to Dong et al., (16%) [17], probably due to the different age of animals used in our study. It should be noted also that the ratio between GluN2A and GluN2B subunits depends on the gender and may be changed in inflammatory conditions [17,18].

In general, our results are consistent with studies by Lee et al., [38], who also found NMDA evoked functional responses in sensory TG neurons. Although these authors did not characterize the response of trigeminal afferent fibers to the specific NMDA agonists, they showed the pro-nociceptive role of NMDA receptors in the development of mechanical hyperalgesia in the masseter muscle [16,38]. Likewise, in our model, the main migraine mediator CGRP, increased the number of TG neurons responding to NMDA. This could be due to the fact that some of NMDA receptors are located in the intracellular pool, since we found that the fraction of functional membrane receptors was lower compared to their total number detected with immunolabelling of GluNA/B subunits.

CGRP is considered as the main endogenous compound that can trigger a migraine attack [32,40]. CGRP peptide and its receptors are expressed in the fraction of TG neurons and their nerve terminals [45,46,47,48]. We previously demonstrated that CGRP had the sensitizing effect on ATP-gated P2X3 [37,48] and 5-HT3 [49,50] receptors involved in activation of trigeminal nociception. Likewise, we show here the increased fraction of NMDA responsive cells after treatment with CGRP. One potential mechanism for this sensitization is that CGRP activates the protein kinase A (PKA) and the protein kinase C (PKC) pathways which promote phosphorylation of NMDA receptors [51]. Consistent with this view, the same PKA/PKC signaling pathways were implicated in CGRP induced sensitization of P2X receptors [48,52]. Peptidergic sensory neurons contain large dense vesicles that act as a reservoir of endogenous CGRP [53]. Potentially, activation of calcium permeable receptors can trigger calcium dependent CGRP release from TG neurons. Indeed, in our experimental model, the classical algogen capsaicin operating via calcium-permeable TRPV1 receptors, promoted the large release of CGRP. Likewise, inflammatory conditions in the meninges can induce significant CGRP release, which was blocked by kynurenic acid, suggesting involvement NMDA receptors in this effect [54]. However, we did not find here the elevation of CGRP release after glutamate or NMDA treatments. This result could be explained by the preferential expression of NMDA receptors in non-peptidergic Aδ fibers characterized by large amplitude of spikes or that the location of NMDA receptors in nerve fibers does not match the location of vesicles with CGRP.

The role of glutamate as a peripheral pro-nociceptive agent in trigeminal system was proposed in several studies [17,21,23,55]. Thus, the peripheral application of glutamate sensitized trigeminal nociceptive afferents innervating deep craniofacial tissues and facial mechanoreceptors [23,55] and activates of masseter muscle afferents [17,21]. However, the involvement of NMDA receptors in the firing of trigeminal afferents innervating meninges was not previously investigated and for first time detected in the current study. This novel finding is consistent with previous observations that monosodium glutamate acting at periphery, increased the ongoing spiking activity in neurons of the spinal trigeminal subnucleus caudalis (SpVc) [19]. Moreover, the expression of GluN2B subunits of NMDA receptors was shown in nerve fibers innervating dural blood vessels [19]. Interestingly, recent study demonstrated the involvement of NMDA receptors in the neurogenic inflammation of the dura mater also suggesting the role of this glutamatergic signalling in initiation of the migraine attack [54].

Currently, peripheral trigeminal nociceptive signalling from meninges is considered as the main mechanism of migraine pain [30,36,56]. We show here that after removal of magnesium, NMDA increases the nociceptive firing of meningeal nerve terminals. This peripheral firing if it happens in vivo conditions, can be transmitted to the higher pain centers to be perceived as a migraine pain. Interestingly, some of these trigeminal terminals were silent in control conditions showing no spontaneous nociceptive activity but they ‘waked up’ after application of NMDA. Interestingly, glutamate and aspartate levels are increased in plasma, cerebrospinal fluid, or platelets during a migraine attack [24,57,58,59] consistent with our finding that both these endogenous aminoacids activated TG neurons. Thus, one hypothesize that these aminoacids can contribute to peripheral nociception acting at trigeminal nerve endings in meninges which re not protected by the blood-brain barrier [60]. In line with this, the high level of glutamate in the diet, was proposed as trigger of headache [34]. In opposite, magnesium supplements in the diet, showed a beneficial protecting effect in fraction of migraine patients [61,62] consistent with its well-known blocking action on NMDA receptors. Notably, magnesium block of NMDA receptors can be reduced not only due to this ion deficiency resulting from inadequate intake or increased gastrointestinal or renal loss [63] but also due to mutations affecting the sites determining action of magnesium and associated with neurological disorders including epilepsy [64]. Such inherited or acquired conditions can promote the excitation of nerve terminal by endogenous glutamate through peripheral NMDA receptors at nerve terminals. While in migraine patients such conditions require compensation of deficiency by magnesium supplements, it is still a matter of debate whether all or only selected patients should get magnesium treatments [65,66]. In view of growing interest to magnesium for migraine treatments, more large-scale clinical trials are needed to clarify this issue.

It is generally accepted that glutamate mediates the fast-synaptic transmission from primary afferents to the second order nociceptive neurons. Thus, in the trigeminal nociceptive system, glutamate which is contained in neurons in small synaptic vesicles can be released by the presynaptic spike to activate AMPA and NMDA receptors located at the postsynaptic nociceptive neurons in trigeminal nucleus caudalis [67] or in dorsal horn neurons [68,69] of the upper cervical segments. NMDA receptors are also implicated as key players in cortical spreading depression (CSD) [70,71,72] underlying migraine aura [73,74,75]. Notably, high extracellular potassium levels, associated with CSD, could be sufficient to remove, via local depolarization, magnesium block of NMDA receptors, thus promote their excitation in primary afferents by endogenous glutamate. Furthermore, the transient relief of the magnesium block of NMDA receptors could be achieved by the other local depolarizing agents such as ATP and 5-HT [33]. The release of endogenous glutamate could be induced by these excitatory compounds or due to antidromic spike propagation from the brainstem [76,77,78]. Together, this novel view is consistent with the concept of the ‘coincidence detector’ function of NMDA receptors, a fundamental phenomenon underlying memory formation [79,80].

## 4. Materials and Methods

Male Wistar P10-12 or P35-40 rats were used. Experimental protocols were performed in accordance with the European Community Council Directive of 22 September 2010 (2010/63/EEC). The protocols were approved by the Animal Care and Use Committee of the University of Eastern Finland (license EKS-008-2019) and the Ethics Committee of Kazan Federal University (protocol No. 8, 5 May 2015).

### 4.1. Cell Culture

Cell cultures were prepared as previously described [81], with modifications. Trigeminal ganglia were isolated from P10-12 Wistar rats and dissociated with the enzymatic cocktail composed of trypsin (0.25 mg/mL, Sigma–Aldrich GmbH, Schnelldorf, Germany) and collagenase type I (760 U/mL, Sigma–Aldrich GmbH) under continuous mixing (850 rpm) at 37 °C for 15 min. Then, the cells were centrifuged (1000 rpm) for 5 min, re-suspended, and plated on pre-coated poly-L-lysine (0.2 mg/mL, Sigma–Aldrich GmbH) coverslips. The cultures were maintained with F12 Nutmix + GlutaMAX medium (Gibco by Life Technologies, Carlsbad, CA, USA) supplemented with heat inactivated fetal bovine serum (FBS, 10%, Gibco Invitrogen) at 37 °C in an atmosphere saturated with 5% CO_2_ for 48 h prior to experiment. Generally, 73 rats were used in cell culture experiments.

### 4.2. Calcium Imaging

Intracellular Ca^2+^ responses were measured by using the fluorescent Ca^2+^ indicator Fluo-3 AM (Life Technologies, Carlsbad, CA, USA). Cells were loaded with 5 µM Fluo-3 AM for 45 min at 37 °C in F12 medium supplemented with FBS 10% and washed once for 10 min with basic salt solution (BSS, Sigma-Aldrich GmbH) containing (in millimolar): 152 NaCl, 3.5 KCl, 10 glucose, 2 CaCl_2_, 10 HEPES at pH 7.40 at room temperature. In each experimental set, magnesium was omitted from the BSS, because magnesium ions block the ion channel of NMDA receptors [25,82]. Fluorescent images were acquired by using an Olympus microscope IX-70, imaging setup (TILL Photonics GmbH, Kaufbeuren, Germany) with a monochromatic light source (excitation wavelength: 488 nm) and a 12-bit CCD camera (SensiCam, Kelheim, Germany). All changes in fluorescence exceeding the flat baseline at the time of agonist application with the characteristic asymmetrical shape (fast rise and slow decay) were considered as Ca^2+^ responses.

NMDA (100 µM, 20 s, Tocris, Bristol, UK), L-aspartate (100 µM, 20 s Sigma–Aldrich GmbH) or L-glutamate (1 mM, 2 s Sigma-Aldrich St. Louis, MO, USA) agonists were applied with co-agonist L-glycine (10 or 30 µM, MP Biomedicals, Solon, OH, USA) in BSS by using a fast perfusion system (Rapid Solution Changer RSC-200, BioLogic Science Instruments, Seyssinet-Pariset, France) followed by the application of KCl (50 mM) to differentiate neurons from satellite glial cells [37] and ionomycin (Abcam, Cambridge, UK), a Ca^2+^ ionophore, to determine maximum calcium response for normalization. DL-2-Amino-5-phosphonopentanoic acid (DL-AP, Tocris Bristol, UK) was used in concentration 80 µM as a specific inhibitor of NMDA receptors.

Cultures exposed to migraine mediator calcitonin gene-related peptide (CGRP, PolyPeptide Laboratories, Strasbourg, France) were pre-incubated for 2 h at 37 °C in F12 medium prior to experiments. Data presented as percentage ΔF/F_0_ were normalized to responses induced by ionomycin (10 µM).

### 4.3. Patch Clamp Recordings

TG neurons were continuously perfused (at ∼2 mL/min) with an external solution containing (in mM): 148 NaCl, 5 KCl, 2 CaCl_2_, 10 HEPES, 10 D-glucose, pH was adjusted to 7.2–7.4 with NaOH. The pipette solution contained (in mM): 135 mM potassium gluconate, 0.1 CaCl_2_, 1 EGTA, 10 HEPES, 2 NaATP, 0.4 NaGTP, osmolarity 290 mOsm, pH was adjusted to 7.2 with KOH. Patch pipettes were prepared from borosilicate glass capillaries (Sutter Instrument, Novato, CA, USA) and had a resistance of 4–5 MΩ. The access resistance (Ra) did not exceed 10 MΩ. NMDA currents were recorded at the holding potential −40 mV in the whole-cell configuration of the patch clamp technique [83]. NMDA currents were evoked by local application of NMDA (100 µM, 2 s) combined with glycine (30 µM) by using a pneumatic picopump (PC-820, WPI, Worcester, MA, USA). Responses to NMDA were measured using Axopatch 200B amplifier (Axon Instruments, Molecular Devices, San Jose, CA, USA). Signals were digitized using an AD-converter (Digidata 1440A, Axon Instruments, Molecular Devices, San Jose, CA, USA) at a frequency of 10 kHz. PClamp 10.3, Clampfit 10.3 (Axon Instruments, Molecular Devices, San Jose, CA, USA) and Origin Pro 2015 (OriginLab Corp., Northampton, MA, USA) programs were used for data acquisition and analysis.

### 4.4. Immunolabeling Staining of NMDA Receptor Subtypes

Cells were fixed with (all reagents from Sigma–Aldrich GmbH): 4% paraformaldehyde, treated with ammonium chloride (0.535 mg/mL), Triton X-100 (0.2%), glycine (15 mg/mL), and bovine serum albumin (BSA, 2%) in phosphate buffer. Cells on coverslips were exposed to primary antibodies anti-β-tubulin III (1:1000, Abcam) combined with primary antibodies either anti-GluN2A (1:1000, Merck, Darmstadt, Germany, or anti-GluN2B (1:1000, Merck) NMDA receptors subunits. After washing to remove primary antibodies, fluorochrome-conjugated secondary antibodies—anti-rabbit Alexa 488 (1:150, Invitrogen, Waltham, MA, USA) and anti-mouse Alexa 633 (1:150, Invitrogen) were added. Before microscopy, coverslips with antibody-bound preparations were pasted on slides with Mowiol glue (Sigma-Aldrich GmbH) to prevent fluorochromes fading. Images were obtained by using Leica SP5 MP (Leica Microsystems Inc., Wetzlar, Germany) confocal microscope with 63× (HCX APO CS 63×/1.4) objective. The fluorescence of Alexa 488 and Alexa 633 was excited using 488 nm and 633 or 568 nm lasers correspondingly. Image capture and processing was made with the Leica LAS AF Software and ImageJ (Leica Microsystems, Wetzlar, Germany) Deconvolution images were obtained using Hyugens Essential Software considering the parameters of confocal imaging at Leica TCS SP5 microscope.

### 4.5. CGRP Level Determination

For measuring CGRP level, an enzyme immunoassay kit (CGRP EIA kit, SPIbio, Montigny Le Bretonneux, France) was used. The samples were collected from trigeminal ganglia primary cultures high density plated on 24 well plates pre-coated with poly-L-lysine, cultured for 48 prior to experiments. Each well plate was gently washed with Dulbecco’s PBS (1×, 500 µL). Then, each well was washed 3 times with 350 µL of magnesium free Hank’s buffered salt solution (HBSS) containing (in millimolar): 5 KCl, 137 NaCl, 19 glucose, 1.3 CaCl_2_, 0.44 KH_2_PO_4_, and 42 NaHCO_3_ at pH 7.4 and continuously bubbled (5% CO_2_: 95% O_2_) for 10 min for stabilization. The wells were filled with 350 µL of vehicle (HBSS, for glutamate or capsaicin test) or glycine (10 µM, for NMDA test) solution for stabilization. Samples were collected (200 µL) after 15 min exposure to the solutions and refilled with fresh solution (200 µL/well) in each well for 15 min for second sample collection. Test compounds (200 µL/well) were added in the final concentrations: glutamate (1 mM), capsaicin (1 µM, Sigma–Aldrich GmbH) or NMDA (30 µ) with glycine (10 µM) for 15 min. Each sample collection was put into Eppendorf tubes with EIA buffer containing peptidase inhibitor and immediately placed in liquid nitrogen. The protocol was carried out in duplicates and following manufacture’s recommendations. Each well provided in the CGRP EIA kit 96-well plate was rinsed 5 times with wash buffer (300 µL/well), CGRP standard and samples (100 µL/well) were allocated as indicated followed by anti-CGRP Ache tracer (100 µL/well) and incubated at 4 °C for 16–20 h. The wells rinsed 3 times with wash buffer (300 µL/well) and incubated with Ellman´s reagent in dark for 45 min at room temperature. The plate was read at 405 nm with a microplate photometer (Wallac VICTOR2™, PerkinElmer, Waltham, MA, USA). The calibration curve was determined by defined standard CGRP concentrations solutions.

### 4.6. Electrophysiology

Hemiskulls for electrophysiological recordings in meningeal preparation were prepared as described by Zakharov et al. [36]. Adult male Wistar rats P35-40 (*n* = 13) were sacrificed by decapitation after CO_2_ inhalation. Skull was cut at sagittal suture level into two halves and brain was gently removed to preserve the structure and expose the dura mater and trigeminal innervation. Isolated hemiskulls were placed on the experimental chamber with BSS containing (in millimolar, Sigma-Aldrich GmbH): 2.5 KCl, 119 NaCl, 18 D-Glucose, 2.7 CaCl_2_, 1 MgCl_2_, 1.1 NaH_2_PO_4_, and 30 NaHCO_3_ at pH 7.4 and continuously bubbled (5% CO_2_: 95% O_2_). As presented by us previously [55,74], for electrical recording, a peripheral branch of meningeal trigeminal nerve was isolated and introduced into a glass electrode. Registration of spontaneous and drug-induced nociceptive signals generated in the trigeminal nerve was performed by using the amplifier DAM 80 (World Precision Instruments, Sarasota, FL, USA). Glutamatergic ligands were applied via fast perfusion (7 mL/min) directed inside of the hemiskull. Recordings of spikes prior to NMDA application and in the presence of this agent were performed in magnesium free solution supplemented with glycine (30 µM). Electrical activity was digitized on a PC using NI PCI6221 board (National Instruments, Austin, TX, USA). Signals were visualized using WinEDR v.3.2.7 software (University of Strathclyde, Glasgow, UK).

### 4.7. Cluster Spike Analysis

Prior spike identification and cluster analysis experimental records were filtered using 100–9000 Hz band-pass Chebyshev type II filter (IIR). Spike detection threshold amplitude normalized to SD of each record was set to five standard deviations (5SD) of base line noise. To obtain standard deviation of baseline recording of 20 s was used. After detection following spike parameters were chosen and documented: (1) positive and (2) negative phase amplitudes; the duration of the (3) positive and (4) negative phase measured at 10% level of positive and negative amplitude respectively; the area of the (5) positive and (6) negative phase. IIR filter, spike detection and analyses were accomplished by custom written program in MATLAB (The MathWorks, Inc., Natick, MA, USA). The choice of parameters for KlustaKwik relied on the ability of 2D parametric plots reveal clear differences in spikes distributions (current amplitude vs. responses). Finally, cluster analysis was done using the KlustaKwik program [34] with the spikes properties such as positive and negative phase amplitudes, the duration of the positive and negative phase and the area of positive and negative phase used as the input parameters for cluster detection. As a result, we could identify 2-18 clusters in each experiment. The number of spikes in each cluster ranged from 5 to 1282.

### 4.8. Data Analysis

Statistical analyses were performed by using Origin 9 (OriginLab Corporation, Northampton, MA, USA), Matlab (The MathWorks, Inc., Natick, MA, USA) and GraphPad Prism 4.0 (GraphPad Software, Inc., San Diego, CA, USA). The Kolmogorov-Smirnov test was used to determine the normality of the data. The two-side Wilcoxon rank sum test or t-test for paired samples were used to evaluate the effect of compounds in the same preparation. Nonparametric Mann-Whitney U test was employed for comparisons between two different groups of samples. Data are expressed as mean ± SEM (standard error of mean). Differences were considered statistically significant at *p* < 0.05, n denotes the number of animals as indicated below.

## 5. Conclusions

In summary, our data suggest the presence of functional NMDA receptors on peripheral sensory trigeminal ganglion neurons, both in somas and in meningeal nerve terminals operating in magnesium dependent manner. These data extend our knowledge on the role of glutamate in the pathophysiology of migraine highlighting potential role of peripheral NMDA mediated mechanisms of this common neurological disorder.

## Data Availability

The data used to support the findings of this study are available from the corresponding author upon request.

## Abbreviations

AMPA: α-amino-3-hydroxy-5-methyl-4-isoxazolepropionic acid; CGRP, Calcitonin gene-related peptide; NMDA, *N*-methyl-D-aspartate; DL-APV, DL-2-Amino-5-phosphonopentanoic acid.
